# Pediatric Familial Hypercholesterolemia: Targeting Intestinal Absorption and Other Therapeutic Strategies

**DOI:** 10.3390/nu17142357

**Published:** 2025-07-18

**Authors:** Konstantinos Arvanitakis, Elena Chatzikalil, Christina Antza, Christos Topalidis, Georgios Kalopitas, Elena Solomou, Vasilios Kotsis, Georgios Germanidis, Theocharis Koufakis, Michael Doumas

**Affiliations:** 1 Division of Gastroenterology and Hepatology, First Department of Internal Medicine, AHEPA University Hospital, Aristotle University of Thessaloniki, St. Kiriakidi 1, 54636 Thessaloniki, Greece; arvanitak@auth.gr (K.A.); gekalopi@auth.gr (G.K.); geogerm@auth.gr (G.G.); 2 Basic and Translational Research Unit, Special Unit for Biomedical Research and Education, School of Medicine, Faculty of Health Sciences, Aristotle University of Thessaloniki, 54636 Thessaloniki, Greece; 3 First Department of Pediatrics, National and Kapodistrian University of Athens Medical School, 11527 Athens, Greece; elenachatz@med.uoa.gr; 4 “Aghia Sofia” Children’s Hospital ERN-PeadCan Center, 11527 Athens, Greece; 5 Third Department of Internal Medicine, Medical School, Aristotle University of Thessaloniki, Papageorgiou Hospital, 56403 Thessaloniki, Greece; kris-antza@hotmail.com (C.A.); vkotsis@auth.gr (V.K.); 6 Department of Pathology, School of Medicine, Aristotle University of Thessaloniki, 54124 Thessaloniki, Greece; topalidi@auth.gr; 7 Department of Internal Medicine, University of Patras Medical School, 26500 Rion, Greece; esolomou@med.upatras.gr; 8 Second Propaedeutic Department of Internal Medicine, Hippokration General Hospital, Aristotle University of Thessaloniki, 54642 Thessaloniki, Greece; thkoyfak@auth.gr

**Keywords:** familial hypercholesterolemia, pediatric patients, gastrointestinal tract, dietary interventions, ezetimibe

## Abstract

Familial hypercholesterolemia (FH) is a genetic disorder marked by significantly elevated levels of low-density lipoprotein cholesterol (LDL-C) since childhood, substantially increasing the risk of premature atherosclerosis and cardiovascular disease. While dysfunction of hepatic LDL-C receptors is the main underlying cause, the gastrointestinal tract plays a key role in cholesterol homeostasis and represents an important therapeutic target. Inhibition of intestinal cholesterol absorption has emerged as an effective strategy in the management of pediatric FH, particularly in patients for whom statins may not be the ideal first-line treatment. Ezetimibe, an inhibitor of the Niemann-Pick C1-like 1 (NPC1L1) protein, has been shown to reduce LDL-C levels in children with FH, with a greater efficacy observed when used in combination with statins. Bile acid sequestrants also enhance cholesterol excretion but are often limited by gastrointestinal side effects, while dietary interventions, such as phytosterol supplementation and fiber-enriched diets, provide additional benefits in lowering LDL-C and are generally well tolerated. Emerging therapies, including microbiota-targeted strategies and novel cholesterol absorption inhibitors, show promise for expanding future treatment options. This review explores the mechanisms of intestinal cholesterol absorption and their relevance to pediatric FH. We examine key pathways, including dietary cholesterol uptake through NPC1L1, bile acid reabsorption, and cholesterol efflux mediated by ATP-binding cassette transporters, while also discussing clinical and experimental evidence on pharmacological and dietary interventions that modulate these pathways. A deeper understanding of cholesterol metabolism, the emerging role of the gut microbiota, and innovative therapeutic agents can support the development of more effective and personalized approaches to the treatment of children with FH.

## 1. Introduction

Cholesterol is a lipid-soluble cyclopentane polyhydrophene derivative of eukaryotic cells, which possesses crucial structural and functional properties [1]. It represents a basic component of the phospholipid bilayer part of the eukaryotic cell membrane, a synthetic precursor of bioactive molecules (e.g., steroid hormones, lipid-soluble vitamins) via oxidative cleavage of side chains, and a crucial regulator of membrane fluidity and signaling pathways as well, via a wide range of protein modification procedures [2,3]. Cholesterol homeostasis, consisting of multiple distinct pathways (endogenous biosynthesis, intestinal absorption, biliary/intestinal reabsorption, and efflux), is of great importance for cellular and systemic functions, considering cholesterol’s interactions with a variety of adjacent molecules and its involvement in enzymatic and non-enzymatic processes [4,5,6]. Moreover, given the multiple vital roles of cholesterol and its metabolism, disruptions in cholesterol homeostasis may lead to severe pathologic adverse events (elevated oxidative stress, persistent inflammatory responses, reduced autophagy, accelerated apoptotic activity), and in some cases, to systemic diseases, including cardiovascular disease, tumorigenic activity, immune system abnormalities, and eye disorders [4,5]. Dysregulation of cholesterol metabolism, congenital or acquired, contributes to disease pathogenesis through several mechanisms related to the different cholesterol metabolic pathways, which can serve as potential therapeutic targets.

**Figure 1:**
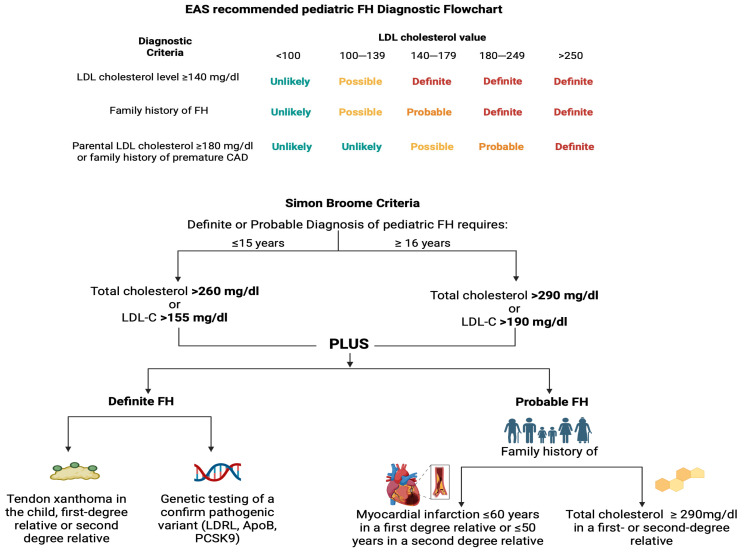
Diagnostic criteria for pediatric FH were established by the European Atherosclerosis Society and the Simon Broome Register Group. The European Atherosclerosis Society criteria include: (i) persistently elevated LDL-C over 140 mg/dL, (ii) family history (parents or siblings) of FH, and (iii) parental history of LDL-C over 180 mg/dL or family history of premature coronary artery disease. Regarding the number and the combinations of these criteria, the diagnosis of FH in a pediatric patient is considered unlikely, possible, probable, or definite. Simon Broome criteria diagnose pediatric FH based on clinical (total cholesterol value, tendon xanthomas), genetic (pathogenic variants: *LDLR*, *ApoB*, *PCSK9*), and familial (myocardial infarction, hypercholesterolemia in a first- or second-degree relative) characteristics. [CAD: coronary arterial disease; EAS: European Atherosclerosis Society; FH: familial hypercholesterolemia; LDL-C: low-density lipoprotein cholesterol]. (The image was created using BioRender software version 04, License #*VI28C5TP5H*).

Familial hypercholesterolemia (FH) is an inherited autosomal dominant or codominant transmitted disorder of lipoprotein metabolism that results in persistent elevation of serum low-density lipoprotein cholesterol (LDL-C) concentrations, being a predisposing factor for premature cardiovascular diseases and, subsequently, premature death [7,8]. FH is the result of genetic alterations in genes encoding proteins involved in the clearance of LDL-C particles. More than 90% of the patients with genetically confirmed FH have mutations in the gene encoding the LDL-C receptor (*LDLR*), and a lower percentage of FH diagnoses are associated with alterations in genes encoding apolipoprotein B (*ApoB*), proprotein convertase subtilisin–kexin type 9 (*PCSK9*), or LDL receptor adaptor protein 1 (*LDLRAP1*) [9]. Heterozygous FH has a frequency of 1 in approximately 300 individuals, while homozygous FH has a frequency of 1 in approximately 250,000–360,000 of the population [10,11]. The diagnosis of FH is based on several sets of criteria (Figure 1), all of which rely on elevated LDL-C levels, usually including clinical features (e.g., arcus cornealis or tendinous xanthomas), family history, and, in some cases, being aided by genetic testing, which is estimated to reveal pathogenic FH mutations in approximately half of pediatric FH patients [12,13]. The European diagnostic criteria of pediatric FH include: (i) elevated persistent LDL-C over 140 mg/dL, (ii) family history (parents or siblings) of FH, (iii) parental history of LDL-C over 180 mg/dL or family history of premature coronary artery disease, after excluding other causes of primary and secondary LDL-C [14]. In cases characterized by difficulties in diagnosis and management, genetic testing should be offered [14]. FH remains a largely underdiagnosed and, therefore, undertreated entity, leading to severe complications in some affected individuals [8]. The main complication of FH is cardiovascular disease, while other serious adverse events, including aortic disease and valvular disease (aortic aneurysm, aortic stenosis, and supravalvular stenosis), have been associated with FH in some cohorts [15].

Considering the arterial functional and morphological deformities that have been described in pediatric patients with FH and the severe complications presented later in the course of the disease, early initiation of treatment is warranted to prevent cardiovascular events in adult life [16]. Current evidence suggests that the induction of lipid-lowering therapy at a young age may decrease the LDL-C burden, improving endothelial functionality, regressing the atherosclerotic events, and ruling out coronary complications in the majority of pediatric cohorts [17,18,19,20]. Established disease guidelines recommend that the treatment of children with FH include a healthy lifestyle with a heart-healthy diet, as well as statin and/or ezetimibe therapy in affected individuals between 8 and 10 years of age, and in younger children only when there is an extreme elevation of LDL-C and the presence of associated risk factors [21,22]; however, continuous research on novel therapeutic targets is currently being conducted. The use of statins in pediatric patients with FH has shown variable response, which differs depending on the specific statin used and, in some cases, the sex of the patient. In female pediatric patients, the use of lovastatin resulted in a 23–27% reduction in LDL-C levels [23], while in male patients the same dose resulted in a 17–24% reduction in LDL-C [24]. Pravastatin has been shown to reduce LDL-C levels to 24.1% from baseline [25], while simvastatin showed a statistically significant decrease (*p* < 0.001, compared to the control group) in LDL-C (41%), TC (31%), apolipoprotein B (APOB) (34%), very low-density lipoprotein (VLDL) cholesterol (21%), and triglycerides (TG) (9%) [26]. Another study comparing the use of rosuvastatin in three doses (5 mg, 10 mg, and 20 mg) versus placebo reported statistically significant changes in LDL-C, TC, and APOB levels for all three doses (*p* < 0.001) [27]. Moreover, a cohort study on statin efficacy in a large pediatric FH population of 214 patients showed a 32% decrease in LDL-C levels (from 6.13 to 4.16 mmol/L), with treatment goals achieved in 20% of the cohort (37 patients) [28]. Data on ezetimibe treatment in pediatric patients with FH are limited. A recent study reported a 22% reduction in LDL-C levels (from 7.3 ± 1.0 mmol/L to 5.7 ± 1.0 mmol/L) after a mean of 105 days of treatment, without significant changes in total cholesterol (TC) or HDL-C levels. The patients remained on ezetimibe for up to 3.5 years, while no medication-related adverse effects were reported [29].

Based on the results of the aforementioned clinical studies, the response of pediatric patients with FH to statins and ezetimibe (measured by reductions in LDL-C levels) does not always achieve treatment targets. Furthermore, evidence from long-term monitoring for adverse events remains limited, underscoring the need for further studies to evaluate the safety of current treatment options throughout the lifetime of FH patients. Future research should also include pediatric patients with secondary forms of dyslipidemia, comparing the outcomes of combination therapy in both inherited and secondary disease groups. As a basis for future research, this review explores the mechanisms of intestinal cholesterol absorption and their relevance to pediatric FH, examining key pathways such as dietary cholesterol uptake via NPC1L1, bile acid reabsorption, and cholesterol efflux mediated by ATP-binding cassette transporters. It also discusses clinical and experimental evidence on pharmacologic and dietary interventions that modulate these pathways. Furthermore, given the implications of the dysregulation of these mechanisms in pediatric patients, we analyze several emerging therapeutic options targeting the gut to reduce LDL-C levels. A deeper understanding of cholesterol metabolism, the emerging role of the gut microbiota, and innovative therapeutic agents can support the development of additional effective and personalized approaches to the treatment of children with FH.

## 2. Pathways Involved in Cholesterol Homeostasis and Basic Aspects of Pediatric FH

### 2.1. Cholesterol Metabolism

#### 2.1.1. Endogenous Biosynthesis

**Figure 2:**
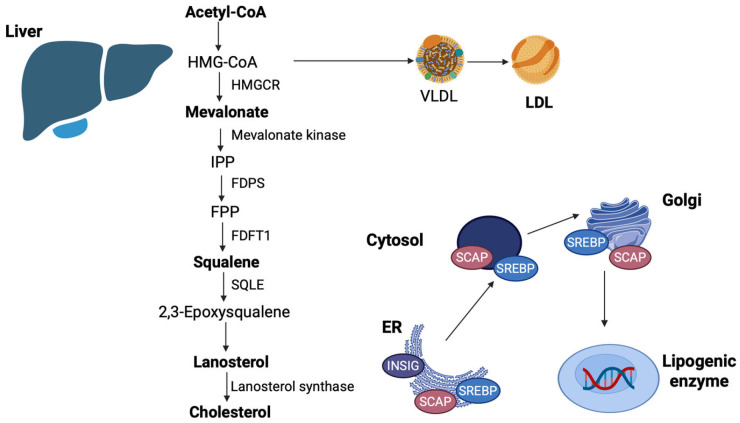
Endogenous synthesis of cholesterol. The key process includes the multiple-step production of lanosterol and cholesterol in the liver. The SCAP protein plays a crucial role as well, enhancing SREBP translocation to the Golgi apparatus, finally leading to the synthesis of lipogenic proteins and sterols, including LDL-C. [FDFT1: farnesyl diphosphate farnesyltransferase 1; FDPS: farnesyl dimethylallyl pyrophosphate synthase; FPP: farnesyl pyrophosphate; HMG-CoA: hydroxy-3-methylglutaryl coenzyme A; HMGCR: HMG-CoA reductase; INSIG: insulin-induced gene protein; IPP: isopentenyl pyrophosphate isomerase; LDL: low-density lipoprotein; SCAP: SREBP cleavage-activating protein; SQLE: squalene epoxidase; SREBP-2: sterol regulatory element-binding protein-2; VLDL: very low-density lipoprotein] (The image was created using BioRender software version 04, License #*SV28G95283*).

Hepatocytes are the main site of cholesterol synthesis using Acetyl-CoA, although smaller amounts are produced by all nucleated cells [5,30]. The biosynthesis of cholesterol is strongly regulated by two major mechanisms of negative feedback, sterol regulatory element-binding protein-2 (SREBP-2) and HMG-CoA reductase (HMGCR) degradation, and consists of four distinct processes: (i) mevalonate (MVA) synthesis; (ii) isopentenyl pyrophosphate (IPP) and dimethylallyl pyrophosphate (DMAPP) production; (iii) squalene synthesis; and (iv) lanosterol formation via squalene cyclization and subsequent cholesterol synthesis [31,32] (Figure 2). MVA undergoes multiple cycles of phosphorylation, decarboxylation, dehydroxylation, and condensation reactions to generate squalene [5]. This squalene is then converted to lanosterol and 27-carbon cholesterol by the action of enzymes, including cyclase and oxygenase, located in the endoplasmic reticulum (ER) [5,30]. SREBP-2 is linked to two protein molecules within the ER membrane: SREBP cleavage-activating protein (SCAP) and insulin-induced gene protein-1 (Insig-1) (Figure 2) [33]. Cholesterol in the ER membrane inhibits the binding of coat proteins to SCAP, preventing the formation of COPII-coated vesicles [34]. A distinct loop region on the SCAP protein, termed methionine-glutamate-leucine-alanine-aspartate-leucine (MELADL), is suggested to regulate the aforementioned mechanism. It is suggested that this SCAP sequence contains multiple domains that are essential for the transport of the SCAP-SREBP complex from the ER to the Golgi apparatus [35]. Cholesterol elimination leads to the dissociation of the SCAP-SREBP protein complex from Insig-1 and to its transportation from the ER to the Golgi apparatus, while Insig-1 is epigenetically modified (mainly ubiquitinated) and subsequently degraded by the proteasome [36] (Figure 2). Elevated cholesterol levels lead to the formation of complexes between cholesterol-derived oxysterols and Insig1/2, which prevents SREBP2 transport to the ER [36].

#### 2.1.2. Intestinal Absorption via NPC1L1

Cholesterol in the intestine is derived from biliary secretion or dietary intake [37]. The intestinal absorption of dietary cholesterol mainly depends on the polytopic transmembrane protein Niemann-Pick C1-Like 1 (NPC1L1), which is expressed both in the apical membrane of the small intestine and in hepatocytes, regulating extracellular sterol transport in the liver, where it inhibits excessive biliary cholesterol loss [38,39]. Intestinal cholesterol absorption, mainly regulated by NPC1L1, consists of three stages: (i) cholesterol solubilization in chylomicrons (CM), (ii) transportation across the apical membrane of enterocytes, and (iii) cholesterol secretion into the bloodstream. Specifically, NPC1L1-dependent cholesterol intestinal absorption is mechanistically regulated by clathrin-mediated endocytosis, and following this, cholesterol is transported to the ER, where it undergoes esterification reactions by acyl-CoA acyltransferase (ACAT), thus participating in the formation of CMs, which are finally secreted into the systemic circulation via the lymphatic system [40,41,42,43]. Lipoprotein lipase (LPL) catalyzes the hydrolysis of CM triglycerides in the peripheral blood, while the remaining CMs bind to the low-density lipoprotein receptor (LDLR) or the LDLR-related protein 1 (LRP) on hepatocyte membranes, enhancing their absorption by liver cells [44]. NPC1L1 is then transported back to the hepatic cell surface, a process that is regulated by several factors, including the microfilament-interacting motor myosin Vb, the small GTPase Rab11a, and the adaptor Rab11-FIP2 [45]. With this mechanism, free cholesterol is dissociated from the NPC1L1 protein during the early stages of endocytosis [45].

Recently, new evidence has emerged supporting the potential of NPC1L1 as a drug target. NPC1L1-mediated cholesterol absorption was fully described in 2020 by a study using cryo-electron microscopy, which suggested that after binding to the sterol-binding specific region, cholesterol triggers the NPC1L1 transporter, changing its formation to enhance the process of cholesterol absorption [46]. Moreover, NPC1L1 can bind different amounts of cholesterol, depending on the different concentrations of cholesterol levels; however, the ability to effectively bind cholesterol lies in its effective dimerization [47]. NPC1L1-mediated cholesterol absorption undergoes esterification by acyl-CoA to form chylomicrons, which are subsequently released into the circulation, where triglycerides are hydrolyzed in order to be used in peripheral tissues or absorbed by hepatocytes [48]. On the other hand, free cholesterol can be pumped back into the intestinal lumen via ATP-binding cassette transporters (ABCs): ABCG5 and ABCG8, or can be released directly into the circulation through ABCA1, in the form of HDL-C [38].

**Figure 3:**
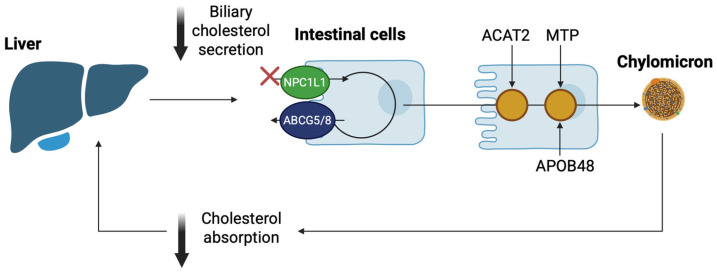
Illustration of the main pathway of cholesterol absorption from the intestinal lumen. High dietary cholesterol, transported via the chylomicron pathway, may lead to excessive cholesterol deposition in the liver, increasing bile cholesterol. Reducing cholesterol absorption in the small intestine, by targeting the NPC1L1 pathway, decreases its availability for secretion into the bile. [ABCG5/8: ATP-binding cassette G 5/8; ACAT2: acetyl-Coenzyme A acetyltransferase; APOB48: apolipoprotein B48; MTP: microsomal triglyceride transfer protein; NPC1L1: Niemann-Pick C1-Like1] (The image was created using BioRender software version 04, License #*SV28G95J5X*).

The effects of targeting intestinal cholesterol absorption via NPC1L1 are illustrated in Figure 3.

#### 2.1.3. Bile Acid Cholesterol Re-Absorption

**Figure 4:**
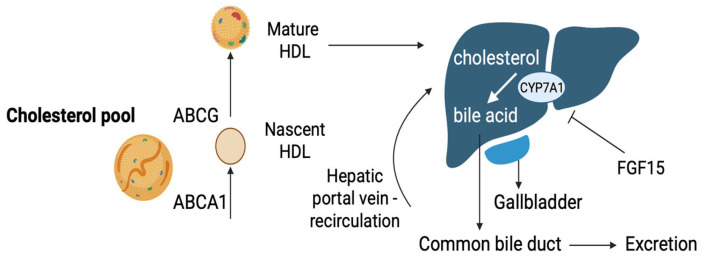
Bile acid cholesterol recycling: the role of ABCG transporters in mature HDL synthesis, the initiation of cholesterol conversion to bile acids via CYP7A1 (inhibited by FGF15), and the fate of bile acids, either being excreted or recirculated via the hepatic portal vein. [ABCA1: ATP-binding cassette A1; ABCG: ATP-binding cassette G; CYP7A1: cholesterol 7α-hydroxylase; FGF15: fibroblast growth factor 15; HDL: high-density lipoprotein] (The image was created using BioRender software version 04, License #*VI28G959BA*).

Hepatocytes play a crucial role in cholesterol downregulation by converting it into bile acids and secreting it into bile [49]. The primary mechanism for cholesterol catabolism is bile acid synthesis, which occurs exclusively in hepatocytes [50]. The enzyme cholesterol 7α-hydroxylase (CYP7A1) initiates this conversion, serving as a rate-limiting step [51]. Bile acids are secreted into bile through the bile salt export pump (BSEP), while cholesterol is transported by the ATP-binding cassette sub-family G member 5 and 8 (ABCG5 and ABCG8) transporters [52] (Figure 4). Most bile acids are reabsorbed by enterocytes through the apical sodium-dependent bile acid transporter (ASBT) and transported back to the liver through the portal circulation [52] (Figure 4). Conjugated bile acids are taken up in hepatocytes by basolateral Na(+)-taurocholate co-transporting polypeptide (NTCP) [52]. Bile acid homeostasis is primarily regulated by feedback mechanisms involving the farnesoid X receptor (FXR), which is found in hepatocytes and intestinal cells, where it reduces CYP7A1 gene transcription through various pathways by the induction of the small heterodimer partner (SHP) and the RNA-binding protein ZFP36L1, which destabilize CYP7A1 mRNA [52,53]. Furthermore, in the intestine, FXR activates fibroblast growth factor 15 (FGF15), which inhibits the expression of CYP7A1 in the liver [52] (Figure 4).

#### 2.1.4. Efflux Through ABC Transporters

Although cholesterol can be produced by almost all cells in the human body, only a few cell types can effectively metabolize it [4]. Reverse cholesterol transport, which refers to the transport of cholesterol to the liver through the systemic circulation to maintain excess cholesterol elimination, can be achieved only by cells that expel elevated cholesterol levels [54]. This complex process is regulated by liver X receptors (LXRs) and the ABC transporters ABCA1 and ABCG1 [55]. LXR represents a ligand-activated nuclear transcription factor that counteracts the effects of the SREBP2 protein, which is activated during cholesterol accumulation [56]. Beyond cholesterol metabolism, LXR represents a crucial regulator of glucose metabolism via increasing the expression levels of glucose transporter 4 (GLUT4) in type 2 diabetes mouse models and modulating inflammatory responses by inhibiting inflammatory factors, primarily NF-κB [57,58].

Human tissues contain two highly homologous LXR proteins that are distributed across various tissues, specifically: (i) LXRα, primarily found in the intestine, liver, kidney, fat tissue, and macrophages, and (ii) LXRβ, which is widely expressed in all human tissues [59]. LRX is heterodimerized in the cell nucleus with the retinoid X receptor (RXR) [4,59]. Binding of the LRX agonist leads to stimulation of target genes including *ABCA1*, *ABCG1*, *SREBP-1c*, and *ApoE* [60]. Regarding LXR downstream targets, ABCA1 and ABCG1, they both use ATP as an energy source to facilitate the transmembrane transport of lipids, sugars, and amino acids [4]. ABCA1 is a full transporter protein expressed throughout the body, characterized by two tandem repeats of transmembrane domains, each containing six segments and a large glycosylated extracellular domain [61]. During the cholesterol efflux process, ABCA1 transfers intracellular cholesterol to lipid-free ApoA1 when activated by LXR, leading to the formation of nascent high-density lipoprotein cholesterol (HDL-C) [4]. Subsequently, lecithin-cholesterol acyltransferase (LCAT) promotes HDL-C maturation. Recent evidence indicates that mutations or inactivation of ABCA1 significantly impede the efflux of cellular cholesterol to ApoA1, as seen in patients with Tangier disease and Abca1-negative mice, who are unable to form mature HDL-C and exhibit low total plasma cholesterol and abnormal lipid deposition in various tissues [62,63]

#### 2.1.5. Cholesterol Balance

**Figure 5:**
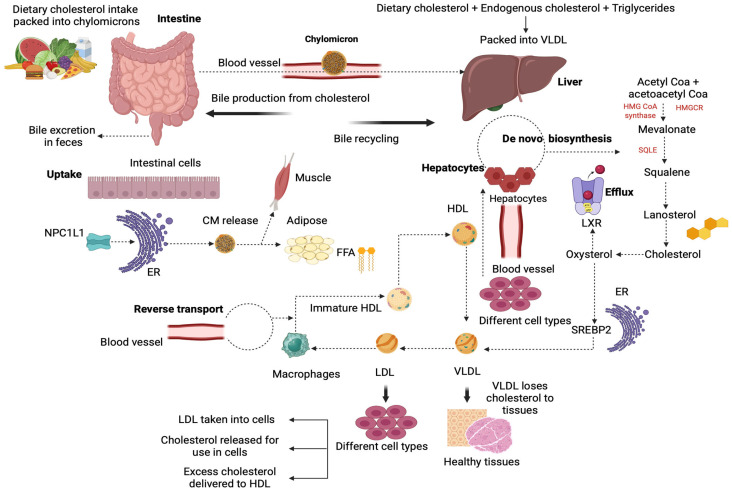
A schematic representation of cholesterol metabolism. Dietary cholesterol is absorbed by the intestine, participating in the assembly of chylomicrons, which are secreted into the blood circulation. After being hydrolyzed, the chylomicron remnants are digested by a low-density lipoprotein receptor on the hepatocyte membrane in order to be absorbed by the hepatocytes. NPL1L1 regulates intestinal cholesterol absorption and further leads to chylomicron release in muscles and adipose tissue. In the liver, cholesterol de novo biosynthesis consists of several enzymatic reactions that convert acetyl-CoA into cholesterol. Sterol regulatory element-binding protein 2 and liver X receptor α are the major regulators of liver cholesterol homeostasis. Reverse cholesterol transport includes transport of free cholesterol to a pre–β high-density lipoprotein particle, generation of mature HDL and VLDL particles through esterification reactions, and cholesterol delivery to the liver through the LDL receptor. LDL-C is taken into hepatic cells, and excess cholesterol is delivered to HDL particles. [ER: endoplasmic reticulum; HDL: high-density lipoprotein; LDL: low-density lipoprotein; LXRα; liver X receptor α; NPC1L1: Niemann-Pick C1-Like1; SREBP2: sterol regulatory element-binding protein 2; VLDL: very low-density lipoprotein]. (The image was created using BioRender software version 04, License #*YE28C5T6LJ*).

Cholesterol homeostasis is regulated by a tight relationship between absorption and synthesis, which is considered crucial to maintaining cholesterol balance in the human body (Figure 5) [5]. Cholesterol synthesis negatively regulates cholesterol absorption and vice versa, maintaining the balance between dietary cholesterol intake and excretion [64]. Studies on cholesterol balance suggest significant variability in individual responses to dietary cholesterol absorption, which is partially explained by the wide variety of protein regulatory mechanisms related to cholesterol handling [64,65]. Increased dietary cholesterol intake increases bile acid synthesis, improving cholesterol excretion through the feces [64]. Moreover, higher cholesterol intake generally reduces endogenous synthesis as a compensatory response, helping to prevent persistently elevated cholesterol levels [66]. Additionally, given that the liver converts cholesterol into bile acids for digestion, which are then reabsorbed in the ileum and returned to the liver, any loss of bile acids (around 5% in feces) is compensated by the ongoing synthesis in the liver, regulated by feedback mechanisms from the reabsorbed bile acids [67].

### 2.2. Basic Aspects of Pediatric FH

Approximately 450,000 children are born with FH each year; however, only about 2.1% of the adult FH population is diagnosed before the age of 18 using current diagnostic methods [68]. Although selective screening based on family history is recommended to detect FH, real-world evidence shows that many cases are missed, in part due to the absence of symptoms related to atherosclerosis during childhood. As a result, most FH cases are discovered incidentally through blood tests during routine pediatric monitoring, following national guidelines [69]. In 2011, the National Heart, Lung, and Blood Institute Expert Panel recommended universal screening for children between 9 and 11 years of age, with a second screening at ages 17 to 21, alongside selective screening, to improve detection of heterozygous FH in the pediatric population [70]. This diagnostic strategy was reaffirmed in the 2018 American College of Cardiology and American Heart Association cholesterol guidelines, which also suggest “cascade screening” as a promising approach [71]. The diagnostic criteria for pediatric FH established in 2017 differ from those in adults in terms of LDL-C thresholds and family history considerations and introduce the category of “probable FH” as previously described [14].

These approaches aim to enable early identification and monitoring of FH in children to reduce missed diagnoses. Following an FH diagnosis, early initiation of lifestyle modifications is recommended. When lifestyle modifications are not sufficient, statin-based pharmacological therapy is considered effective in lowering LDL-C levels and is safe in the short and medium term, reducing disease-related complications [72]. Additional lipid-lowering medications can be used in combination with statins in patients for whom statin monotherapy is inadequate, particularly in homozygous FH patients that often require aggressive multimodal treatment [73]. If left untreated, FH is associated with an increased risk of premature coronary artery disease. Several predictive markers have been proposed, including combinations of clinical and biochemical markers, genetic risk scoring systems, inflammatory markers, and imaging techniques (e.g., carotid intima-media thickness and coronary artery calcium scoring) [74]. However, these predictive markers have not yet demonstrated sufficient efficacy for widespread use in daily clinical practice. Therefore, more studies are needed to refine diagnostic and prognostic algorithms, determine the optimal timing and intensity of statin therapy, and establish the long-term safety and outcomes of lipid-lowering treatments in pediatric cohorts with FH.

## 3. Therapeutic Options for Pediatric FH

Recent studies indicate that the pathophysiological atherosclerotic changes in individuals with FH occur early in their course of the disease; therefore, therapeutic interventions in childhood aiming to reduce LDL-C levels may be important to prevent FH complications, mainly cardiovascular disease [18]. Statins are the first-line medications for FH in pediatric patients, while ezetimibe can be induced at the age of 8–10 years for heterozygous FH patients and from diagnosis for homozygous FH patients [75]. However, even in cases of early induction of established therapeutic choices, guideline-recommended LDL-C cholesterol levels cannot be achieved in some pediatric FH patients; thus, we should suggest the use of novel therapeutic agents, which mainly target the intestinal cholesterol absorption [14,76].

### 3.1. Established Therapeutic Options for Pediatric FH and Ezetimibe Mechanism of Action

Regarding the established therapeutic options of pediatric FH, the European Atherosclerosis Society and the American College of Cardiology (American Heart Association) have proposed several guidelines that indicate the importance of lifestyle changes in combination with the initiation of statin therapy, from 8 to 10 years of age for FH heterozygotes and from the time of diagnosis for FH homozygotes [75,77]. Balanced dietary intake, regular physical activity, medical interventions for female adolescents with dyslipidemia (birth control), and monitoring for common risk factors (smoking, obesity, and diabetes) are considered key components of successful long-term pediatric FH management, decreasing the risk of developing severe complications, especially in patients with homozygous FH [28]. The 2017 guidelines for dyslipidemia suggest a fat-energy ratio of 20–25% and a carbohydrate-energy ratio of 50–60% for pediatric patients with FH, according to the indications for the adult population [78]. In pediatric patients with LDL-C levels equal to or above 180 mg/dL, pharmacotherapy is suggested, including statins as the first-line treatment and ezetimibe as the second-line treatment [78].

Serum cholesterol levels are regulated through a dynamic relationship between cholesterol biosynthesis in the liver and intestinal absorption [79]. Statins are competitive inhibitors of the HMG-CoA reductase enzyme and decrease serum LDL-C by inhibiting cholesterol biosynthesis, leading to elevated expression of the LDL receptor, thus facilitating the removal of LDL-C from the systemic circulation. Inhibition of LDL-C biosynthesis decreases total cholesterol expression in the ER, resulting in the transportation of SREBPs from the endoplasmic reticulum to the Golgi apparatus and subsequent cleavage into active transcription factors [80,81]. SREBPs translocate to the nucleus, elevating the expression of several genes, including HMG-CoA reductase, restoring liver cholesterol biosynthesis and the number of LDL receptors on the plasma membrane of hepatocytes, further resulting in increased clearance of LDL, thus reducing LDL-C levels [82,83]. In homozygous FH, which is characterized by the total absence of normally functioning LDL receptors, statin monotherapy is not considered effective in decreasing LDL-C levels [79].

The efficacy of using statins in decreasing LDL-C levels in FH pediatric patients has not been established yet, while long-term evidence on the effect of statin therapy in protecting against disease complications is currently lacking, especially when compared to adults, for whom the importance of statin treatment in the prevention of cardiovascular disease is based on robust data [84,85,86]. Luirinik et al. reported a 20-year follow-up study in FH pediatric patients, who were followed up alongside their unaffected healthy siblings in terms of the therapeutic results of statin use and alongside their affected parents regarding the incidence of clinical cardiovascular disease [28]. The mean levels of LDL-C reduced by 32% from baseline, while the mean progression of carotid intima—median thickness, which was used to evaluate the effect of statins, was similar between the two groups (patients and healthy siblings) during the study period [28]. The incidence of cardiovascular adverse events and cardiovascular-related death was significantly lower in patients with FH at 39 years of age compared to their FH counterparts who started statin use later in the course of the disease [28]. This study, which is, to the best of our knowledge, the largest one to evaluate statin use in pediatric FH patients, suggests that early initiation of statins in this population is safe and effective and reduces the long-term risk of cardiovascular complications [28].

**Figure 6:**
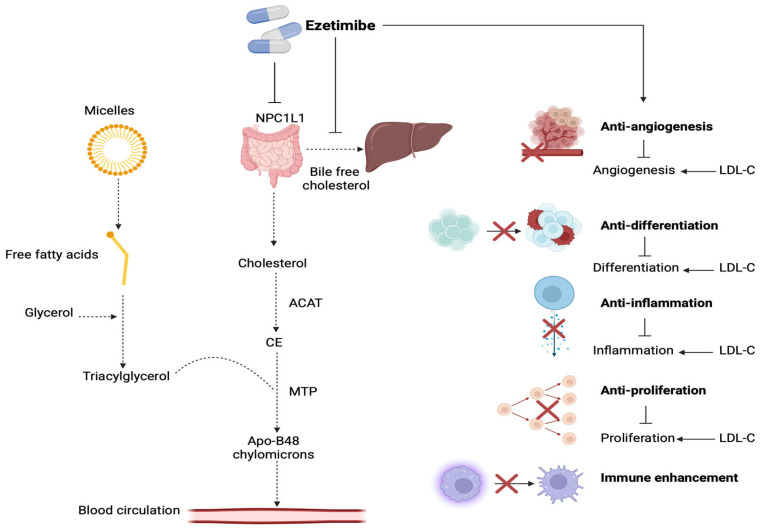
Illustration of the mechanism of action and therapeutic properties of ezetimibe. Ezetimibe inhibits cholesterol absorption by directly attaching to a specific transmembrane domain of the NPC1L1 protein. Therefore, internalization of the NPC1L1/cholesterol complex is inhibited, preventing cholesterol from entering the enterocyte. This blocks the formation of initial Apo-B48 chylomicrons (through the ACAT/MTP pathway) that normally enter the bloodstream, and, as a result, LDL-cholesterol levels are decreased. Ezetimibe presents anti-inflammatory and anti-tumorigenic (antiangiogenetic, antiproliferative) properties, as well as reducing the risk of hypercholesterolemia complications. [ACAT: acyl-coenzyme A: cholesterol acyltransferase; CE: cholesterol esters; LDL: low-density lipoprotein; MTP: microsomal triacylglycerol transfer protein; NPL1L1: Niemann-Pick C1-Like1]. (The image was created using BioRender software version 04, License #*VF28C5SX19*).

Ezetimibe is an NPC1L1 inhibitor, representing an effective treatment option to decrease LDL-C cholesterol levels [41,87]. Ezetimibe inhibits caveolin-1 and annexin-2, decreasing intestinal cholesterol absorption and reducing liver cholesterol levels [88]. Specifically, ezetimibe prevents cholesterol absorption by directly binding to a transmembrane loop for NPC1L1, inhibiting its ability to bind with clathrin/AP2 that is required for its endocytosis, by which cholesterol is released into the cytosol and is esterified by cholesterol acyltransferase (ACAT) to form cholesterol esters [89,90]. These cholesterol esters, combined with triacylglycerol particles by facilitation by microsomal triacylglycerol transfer protein (MTP), form ApoB-48 containing chylomicrons, which will be taken up in the systemic circulation [91,92]. Ezetimibe inhibits this mechanism, selectively affecting cholesterol without impacting the absorption of triglycerides, enhancing the expression of liver LDL receptor levels, and resulting in an 18–20% decrease in circulating LDL-C levels [93]. Ezetimibe is metabolized in the liver and intestine, has a long half-life of 22 h, and is predominantly excreted in the feces while minimally interacting with medications metabolized by cytochrome P450 [94]. In particular, ezetimibe, by decreasing LDL-C levels, inhibits the inflammatory and tumorigenic effects of excess LDL-C in the systemic circulation, presenting antiangiogenetic, antiproliferative, and immune regulatory properties (Figure 6) [95].

Although statin monotherapy has been shown to decrease LDL-C levels, it stimulates intestinal cholesterol absorption, which is considered a limiting factor in its efficacy [96]. Combined treatment with statins and ezetimibe shows a significant synergistic effect in decreasing LDL-C levels in pediatric patients, while recent evidence suggests ezetimibe monotherapy as a safe and effective therapeutic option [97,98,99]. A prospective study in pediatric patients with FH, who received ezetimibe monotherapy at 10 mg/day for a median time of 15.9 months, showed a significant decrease (315.3 +/− 41.8 to 233.3 +/− 36.8 mg/dL) in total cholesterol and LDL-C levels. Their triglyceride levels and biochemical profile remained stable, and no adverse events were observed in this cohort [100]. Regarding combinational treatment schemas, a recently published meta-analysis of 41 studies including 4667 children with heterozygous HF and a median age of 12 years reported a 33.44% median reduction in LDL-C levels with statin and ezetimibe co-administration [101]. Moreover, a registry analysis of 11,848 FH pediatric patients revealed that only 28.5% were on lipid-lowering treatment during the course of the disease and that median LDL-C levels were higher in children not receiving treatment as compared to their counterparts under lipid-lowering treatment [102].

### 3.2. Novel FH Therapeutic Strategies Targeting Intestinal Cholesterol Metabolism

The therapeutic options previously analyzed, namely statins and ezetimibe, are dependent on LDL receptors, demonstrating limited efficacy in patients with homozygous FH variants, who do not reach the LDL-C levels recommended by the guidelines [103,104,105,106]. Lipoprotein apheresis is an alternative therapeutic option indicated for patients with early-onset FH, which can effectively decrease LDL-C levels by 50–70%. However, this decrease is temporary, and each treatment, which is necessary every 1–2 weeks, is time-consuming and affects the quality of life of patients [107,108]. Considering that there is a percentage of pediatric FH patients who do not reach target LDL-C levels despite treatment, the need to establish new therapeutic goals remains undeniable, especially in individuals who respond poorly to conventional therapies, even when receiving the maximum tolerated doses of first-line and second-line medications. Furthermore, given the efficacy of ezetimibe in children with FH, current research has focused on novel drugs that target intestinal cholesterol absorption. In this context, recent advances in FH drugs acting on the intestinal tract have surfaced, and several new treatment options are currently being investigated in randomized clinical trials [109]. It is also worth noting that other treatment alternatives targeting angiopoietin-like 3 (ANGPTL3) have emerged as candidates for FH treatment, especially for individuals with refractory FH [110]. ANGPTL3 inhibitors, namely evinacumab, promote the excretion of the very low-density lipoprotein before it can be converted to LDL-C [111]. In the following paragraphs, we focus on drugs that target intestinal cholesterol absorption in pediatric patients with FH.

Novel medications for pediatric FH mainly include PCSK9 inhibitors. PCSK9 primarily regulates LDL-C levels by targeting and degrading LDL receptors (LDLR) [112]. This process includes the interaction of PCSK9 with LDLR’s EGF-A domain, directing the LDL-C/LDLR complex to lysosomes for degradation, thus reducing LDLR on cell surfaces and leading to increased circulating LDL-C [112]. PCSK9 is expressed in the liver and small intestine, with hepatic PCSK9 playing a dominant role in cholesterol regulation [113]. Current research indicates that intestine-specific PCSK9 has less impact on lipidemia. Additional roles of PCSK9 include involvement in hepatic inflammation; inflammatory stimuli increase PCSK9 expression, decreasing LSLR in the liver and promoting inflammation through macrophage activation and cytokine production. This creates a cycle that worsens liver inflammation through the NLRP3 inflammasome and TLR4/NFκB signaling pathways [114]. Evolocumab is a monoclonal antibody targeting intestinal cholesterol absorption and specifically pro-protein convertase subtilisin-kexin type 9 (PCSK9) expression in the small intestine, preventing PCSK9 from degrading the LDL-C receptor, thus increasing LDL-C receptor availability and subsequently decreasing LDL-C levels up to 60%, according to current real-world data [115]. It is worth noting that PCSK9 interacts with CD36, a platelet glycoprotein that enhances LDL-C uptake, leading to decreased lipid uptake, increased inflammation, and accelerated progression of atherosclerosis [116]. Therefore, PCSK9 inhibitors can decrease LDL-C as well as thrombosis markers in patients with FH, preventing arterial clotting events, including heart attacks and ischemic strokes [116].

**Figure 7:**
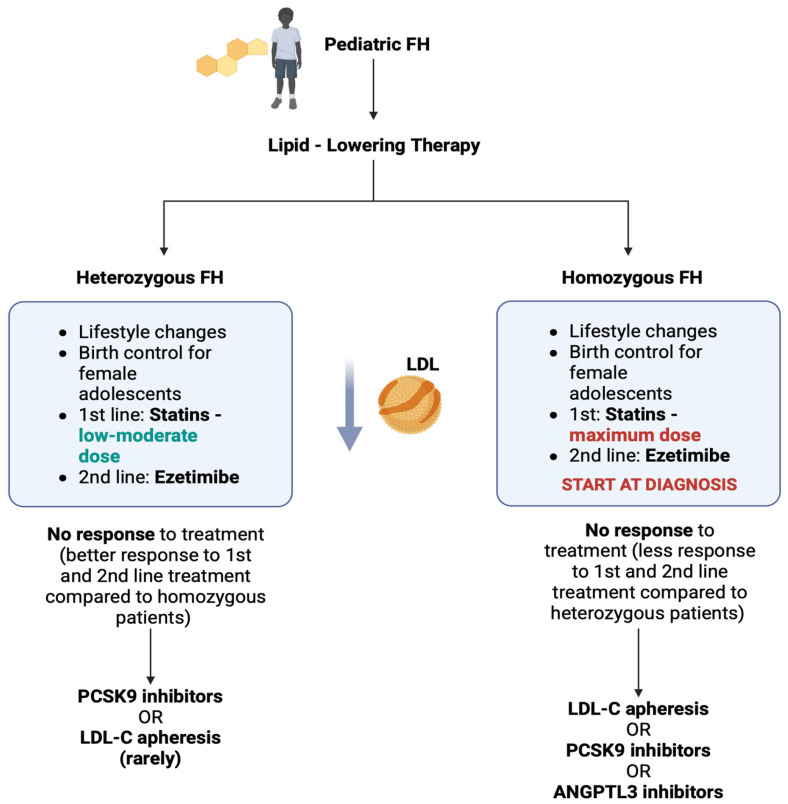
Algorithm for the therapeutic evaluation of pediatric familial hypercholesterolemia in homozygous and heterozygous patients. Lifestyle changes, as well as 1st and 2nd line treatment (statins and ezetimibe, respectively), apply both to homozygotes (maximum statin dose) and heterozygotes (low to moderate statin dose). In cases of non-response to treatment, which are more common in homozygous FH patients due to the absent or significantly defective LDL receptor activity, LDL-C apheresis and the use of PCSK9 inhibitors should be considered. [ANGPTL3: angiopoietin protein-like 3; FH: familial hypercholesterolemia; LDL-C: low-density lipoprotein cholesterol; PCSK9: proprotein convertase subtilisin kexin9]. (The image was created using BioRender software version 04, License #*MD28C5S0B7*).

PCSK9 inhibition was initially approved in 2015 for adult patients with hyperlipidemia and in 2017 for people with cardiovascular disease and increased risk for myocardial infarction, stroke, and revascularization in atherosclerotic cardiovascular disease [117,118,119,120]. PCSK9 inhibitors, namely evolocumab and alirocumab, can reduce LDL-C levels by approximately 20–25% and 55–60% in homozygous FH patients and people with severe heterozygous FH, respectively [110]. Considering that the intestine influences the overall lipid levels via lipid absorption and that the direct effects of PCSK9 inhibition are centered on hepatic pathways (as hepatic PCSK9 plays the major role in cholesterol regulation), targeting PCSK with novel medication, including evolocumab, alirocumab, and inclisiran, is a promising option for non-responders to 1st and 2nd line FH treatment [116]. In pediatric cohorts, evolocumab and alirocumab have been shown to be effective and well-tolerated [110] and are recommended for FH pediatric patients ≥12 years of age who cannot tolerate statins as monotherapy or in combination with other agents or fail to respond to conventional treatment (ezetimibe) [101]. Furthermore, the liver production of PCSK9 has not yet been targeted by inclisiran, a gene-suppressing drug that is effective in adults but is still being investigated for pediatric use [121]. A summary of FH therapeutic options for the pediatric population, including intestinal absorption-targeting medication, is illustrated in Figure 7.

Only a limited number of clinical studies have evaluated the use of PCSK9 inhibitors in pediatric patients with FH. In more detail, six studies have investigated the efficacy of evolocumab or alirocumab on LDL-C levels in pediatric cohorts of FH, and the results on therapeutic efficacy demonstrated great variability, which may reflect the variability of LDL receptor activity as a result of the heterogenous genetic profile of the patients. Homozygous FH was subcategorized into three types, namely defective/defective, defective/null, and null/null, according to whether the activity of the LDL receptor in each allele was null or defective [118,122,123,124,125,126,127]. Real-world evidence on alirocumab [128] and evolocumab, obtained from the RAMAN and HAUSER-OLE studies, suggests using PCSK9 inhibitors to achieve a reduction equal to or above 15%, translating into an absolute reduction of >60 mg/dL, for the treatment of pediatric FH [129]. Two other clinical trials, namely TESLA and TAUSSIG, which evaluated 15 homozygous children with FH in total, indicate the use of evolocumab in these cohorts [125,128]. Evolocumab had a great safety profile, with no adverse events reported, while LDL-C levels were significantly reduced, as well as other variables of the lipid profile, including apolipoprotein B and lipoprotein-a [125,128]. Regarding heterozygous pediatric FH, the phase 2 ODYSSEY KIDS study, involving 42 pediatric patients, reported a significant decrease in LDL-C levels within 8 weeks of alirocumab induction [130]. Along the same line, the HAUSER-RCT phase 3 clinical trial evaluated the efficacy and safety of evolocumab in combination with statins with or without ezetimibe in 157 pediatric heterozygous FH patients in whom LDL-C guideline-approved levels had not been achieved, reporting a median decrease of 77.5 mg/dL in LDL-C levels [131], while no clinically relevant effects of evolocumab on steroid hormone biosynthesis and vitamin (A, D, E, and K) levels were observed [131]. (Table 1.)

## 4. Discussion and Future Perspectives

The purpose of the FH therapeutic intervention is to reduce the cumulative burden of persistently elevated LDL-C levels and, at the same time, to prevent the development of complications, especially cardiovascular adverse events [130]. Effective monitoring and control of LDL-C has been associated with a significant reduction in morbidity and mortality in FH patients, highlighting the importance of lipid-lowering treatment, which is suggested to be administered early in the course of the disease [132]. Statins represent the mainstay of FH treatment, especially when combined with a low-saturated fat diet, which lowers cholesterol and improves cardiovascular outcomes [133]. The recommended statin dose in the pediatric population is lower compared to adults; however, the majority of children diagnosed with FH receive statin monotherapy (first-line treatment), with or without ezetimibe (second-line treatment), without the need for additional medication. Unfortunately, some pediatric patients do not reach the LDL-C levels recommended by the guidelines, mainly due to limited drug response or poor treatment adherence [133]. To overcome the non-response in these cases, a slew of novel medications that aim to reduce LDL-C levels in severe cases, mostly with combinational lipid-lowering regimens, are being tested in ongoing clinical trials.

The cholesterol pool in the human body is derived from both biosynthesis and dietary uptake. Although statin monotherapy has been proven to be effective in reducing cholesterol biosynthesis, it lacks the ability to inhibit the absorption of cholesterol; this point is where current research is directed, providing novel treatment options targeting intestinal cholesterol absorption. Evolocumab and alirocumab are the new intestine-targeting drugs evaluated in pediatric FH cohorts, presenting promising effects in reducing LDL-C levels, with most studies suggesting a median decrease of 15% in half of patients [129]. It is worth noting that the response depends on the genetic profile of pediatric patients with FH, since homozygotes respond poorly to PCSK9 inhibition compared to their heterozygote peers. However, the effects of evolocumab and alirocumab can be safely evaluated after their implementation in large cohorts, with appropriate adaptation to the genetic heterogeneity of pediatric patients and/or differences in existing treatment [132]. It is, however, suggested that PCSK9 inhibitors represent a viable therapeutic option for homozygous FH children who are not responding adequately to high-intensity statin and ezetimibe therapy; if at least a 15% additional decrease in LDL-C levels is observed, PCSK9 inhibitor therapy may be continued, otherwise, this therapy is discontinued [107]. Familial hypercholesterolemia in pediatric cohorts is associated with a significant risk of cardiovascular complications; therefore, early recognition and timely therapeutic evaluation are considered necessary to achieve an optimal prognosis. Many pediatric patients with FH do not benefit from first-line statin treatment alone, nor from second-line ezetimibe-targeted treatment.

Recently developed monoclonal antibodies targeting the gut can be considered additional lipid-lowering therapeutic options, as recommended by ongoing clinical trials. Evolocumab and alirocumab, targeting PCSK9, have been shown to be effective in significantly reducing LDL-C levels in the pediatric population with FH. However, more research is needed on their long-term safety and efficacy, as evidence on long-term monitoring, especially for FH homozygotes, currently remains limited. The most promising future research directions for pediatric and, in particular, homozygous FH patients include inhibitors of cholesterylester transfer protein (CETP) (obicetrapib), RNAi agents (ARO-ANG3, olpasiran), antisense oligonucleotides, and emerging therapies such as ASGR1 inhibitors and HMGCR degraders, all of which aim to reduce serum LDL-C, with subsequent improvement in cardiovascular health of children.

The mainstay of treatment continues to be statins, often in combination with ezetimibe, PCSK9 inhibitors, or newer agents such as inclisiran and bempedoic acid. For homozygous FH, therapies such as lomitapide and evinacumab offer hope to lower lipids in refractory cases. Despite the availability of these options, the real-world adherence to therapy and the achievement of LDL-C targets remain suboptimal. Public health strategies must prioritize education, early detection, and access to advanced therapies to mitigate the long-term cardiovascular burden of FH. Additionally, emerging therapeutic approaches, including gene-based treatments, hold promise for a more definitive correction of the underlying genetic defect in the future. Continued research is essential to refine diagnostic algorithms, personalize therapy based on genotype-phenotype correlations, and develop cost-effective screening models. Multidisciplinary collaboration between clinicians, researchers, and policymakers will be crucial to overcoming current challenges and improving the prognosis of individuals with FH. Ultimately, a combination of early identification, aggressive lipid management, and innovations in therapy will be the key to reducing the global impact of this inherited lipid disorder.

Based on our daily clinical experience, pediatric patients with homozygous familial hypercholesterolemia are typically closely monitored to prevent and effectively manage cardiovascular complications related to the disease. The diagnosis of homozygous FH is most often prompted by the presence of xanthomas and total cholesterol levels exceeding 240 mg/dL. Until about two decades ago, homozygous FH was associated with high early mortality rates due to cardiovascular complications. The absence of effective, established medications made survival particularly difficult for these patients, often resulting in psychological distress and unnecessary adoption of extremely restrictive diets, which alone could not sufficiently improve their health. Current clinical practice in our country recommends a healthy diet in combination with pharmacologic treatment, primarily statins, for patients with homozygous FH in order to control lipid levels and reduce cardiovascular risk. Statin therapy has significantly improved clinical outcomes and quality of life for our patients; however, the introduction of novel agents could further enhance health outcomes and help eliminate disease-related complications. In general, the use of appropriate diagnostic algorithms to effectively detect and prevent severe forms of FH, together with the implementation of effective strategies to manage cardiovascular complications, are key components of our routine clinical care for patients with homozygous FH.

## 5. Conclusions

Pediatric FH represents a critical and often overlooked challenge in cardiovascular prevention. Early onset of elevated low-density lipoprotein cholesterol levels in children with FH substantially increases the lifetime risk of premature atherosclerosis and cardiovascular disease. Despite advances in lipid-lowering therapies and increasing awareness, the condition continues to be underdiagnosed and undertreated, especially in its heterozygous and homozygous forms. The complex genetic underpinnings of FH-ranging from monogenic to polygenic causes-highlight the need for comprehensive diagnostic approaches that combine clinical criteria with genetic testing. Early detection, ideally through cascade screening of family members, is crucial to initiating timely and effective interventions, and current guidelines recommend the initiation of lifestyle modifications and statin therapy in childhood. However, many children do not reach the LDL-C target levels with conventional therapies alone, which underscores the need for complementary and alternative strategies. A central focus of current research is the role of the gastrointestinal tract in cholesterol homeostasis and its growing therapeutic relevance. Intestinal cholesterol absorption, largely mediated by the NPC1L1 transporter, has emerged as a promising target to reduce LDL-C in pediatric FH. Ezetimibe, a selective inhibitor of NPC1L1, has been shown to be safe and effective in reducing LDL-C levels in children, both as monotherapy and in combination with statins. In addition, emerging treatments such as PCSK9 inhibitors and other agents targeting intestinal mechanisms present additional options for children who are statin-intolerant or are not adequately controlled with existing therapies. Understanding and modulating intestinal cholesterol absorption not only complements hepatic-targeted therapies but also opens new avenues for personalized care in pediatric FH. Future research should continue to refine these strategies, exploring age-specific pharmacodynamics, long-term safety, and the role of the gut microbiome. Early, targeted intervention-especially through intestinal pathways-offers a compelling opportunity to alter the natural course of FH and significantly improve cardiovascular outcomes for affected children.

## Figures and Tables

**Table 1:** Novel therapeutic strategies targeting intestinal cholesterol metabolism for the treatment of pediatric FH.

Clinical Studies/Trials	Medication	Target	Mechanism of Action	% Reduction in LDL-C	Efficacy in Pediatric FH	Serious Adverse Events
Approved [117,118,119,120]	Evolocumab	PCSK9	Prevents PCSK9 from degrading LDL-C receptor, increasing LDL-C receptor availability	20–60%	Efficient and approved for pediatric FH from 12 years of age	Not reported
ODYSSEY KIDS [130]	Alirocumab	PCSK9	Prevents PCSK9 from degrading LDL-C receptor, increasing LDL-C receptor availability	21–62%	Efficient and off-label used for pediatric patients above 8 years of age with severe FH	Not reported
ORION-16 [121]	Inclisiran	PCSK9	PCSK9 gene-silencing medication	35–43%	Evaluated in pediatric FH in the ORION-16 study	N/A

[FH: familial hypercholesterolemia; LDL-C: low density lipoprotein cholesterol; PCSK9: proprotein convertase subtilisin kexin 9].

## Data Availability

No new data were created.

## Abbreviations

The following abbreviations are used in this manuscript:

CAD	coronary arterial disease
EAS	European Atherosclerosis Society
FH	familial hypercholesterolemia
LDL-C	low-density lipoprotein cholesterol
ANGPTL3	angiopoietin protein like 3
PCSK9	proprotein convertase subtilisin kexin-9
ACAT	acyl-coenzyme A: cholesterol acyltransferase
CE	cholesterol esters
LDL	low-density lipoprotein
MTP	microsomal triacylglycerol transfer protein
NPL1L1	Niemann-Pick C1-Like1
ER	endoplasmic reticulum
HDL	high-density lipoprotein
LXRα	liver X receptor α
SREBP2	sterol regulatory element-binding protein 2
VLDL	very low-density lipoprotein
LDLRAP1	LDL receptor adaptor protein 1
HMGCR	HMG-CoA reductase
MVA	mevalonate synthesis
IPP	isopentenyl pyrophosphate dimethylallyl pyrophosphate
DMAPP	dimethylallyl pyrophosphate
CETP	cholesterylester transfer protein
LCAT	lecithin-cholesterol acyltransferase
RXR	retinoid X receptor
LPL	lipoprotein lipase
MELADL	methionine-glutamate-leucine-alanine-aspartate-leucine
GLUT4	glucose transporter 4
SCAP	SREBP cleavage-activating protein
Insig-1	insulin-induced gene protein-1
